# Extracorporeal Circulation and Optic Nerve Ultrasound: A Pilot Study

**DOI:** 10.3390/medicina59030445

**Published:** 2023-02-23

**Authors:** Öztürk Taşkın, Ufuk Demir

**Affiliations:** Department of Anesthesiology and Reanimation, Kastamonu University Faculty of Medicine, 37150 Kastamonu, Turkey

**Keywords:** optical nerve sheath diameter, cardiopulmonary bypass, intracranial pressure, extracorporeal circuit

## Abstract

*Background and Objectives*: Cardiopulmonary bypass (CPB) is an extracorporeal circuit that provides surgical access to an immobile and bloodless area, allowing for technical and procedural advances in cardiothoracic surgery. CBP can alter the integrity of the blood–brain barrier and cause changes in intracranial pressure (ICP) postoperatively. Optical nerve sheath diameter (ONSD) measurement is among the alternative non-invasive methods for ICP monitoring. In this study, we aimed to evaluate the optic nerve sheath diameter measurements under the guidance of ultrasonography for ICP changes during the extracorporeal circulation process. *Materials and Methods*: The study population included 21 patients over 18 years of age who required extracorporeal circulation. Demographic data of the patients, such as age, gender, comorbidity, American Society of Anesthesiologists (ASA) classification and reason for operation (coronary artery disease or mitral or aortic valve disease) were recorded. The ONSD was measured and evaluated before the extracorporeal circulation (first time) and at the 30th minute (second time), 60th minute (third time) and 90th minute (fourth time) of the extracorporeal circulation. Non-invasive ICP (ICP ONSD) values were calculated based on the ONSD values found. *Results*: The mean ONSD values measured before the extracorporeal circulation of the patients were found to be 4.13 mm (3.8–4.6) for the right eye and 4.36 mm (4.1–4.7) for the left eye. Calculated nICPONSD values of 11.0 mm Hg (1.0–21.0) for the right eye and 10.89 mm Hg (1.0–21.0) for the left eye were found. It was observed that there was a significant increase in the ONSD and nlCPONSD values recorded during the extracorporeal circulation of all patients compared to the baseline values (*p* < 0.005). *Conclusions*: During extracorporeal circulation, ultrasound-guided ONSD measurement is an easy, inexpensive and low-complication method that can be performed at the bedside during the operation to monitor ICP changes.

## 1. Introduction

In the first years of cardiac surgery, many open-heart surgeries were performed on beating hearts. However, as a result of studies, CPB and cardioplegia techniques have been developed, and now, most coronary bypasses are performed using a heart–lung machine [1]. Although coronary bypass surgery that is performed on a beating heart without extracorporeal circulation is more advantageous in terms of the fewer complications that may occur, it is not preferred by much. Coronary artery bypass surgery using extracorporeal circulation is the procedure that is mainly applied in many centers in cardiovascular surgery today. The CPB technique is actively used today and can cause various complications in some tissue and organ functions in the body. However, there is not yet a perfusion system that does not cause any adverse effects on the hemodynamics of the body, maintains routine physiological balances, provides the complete tissue perfusion of all organs, especially the brain, and does not cause any hemolysis in blood elements [2,3]

Cardiopulmonary bypass (CPB) is an extracorporeal circuit that allows technical and procedural advances in cardiothoracic surgery and provides surgical access to an immobile and bloodless area [4]. Adverse neurological outcomes may occur after cardiac surgery and CPB despite improvements in anesthesia and surgery [5]. After open-heart operations, the risk of cognitive dysfunction and stroke increases in direct proportion to age. Since cardiac operations are being applied to older patients today, surgical complications are more common. After open-heart operations, symptoms such as transient ischemic attack and stroke are observed in 1–3%, and complications such as temporary cognitive dysfunction and seizures are observed in 5–10% [6].

Hypoxic ischemic events, embolism, changes in the blood–brain barrier and increased intracranial pressure (ICP) are among the causes of these adverse outcomes [7,8]. CBP may alter the integrity of the blood–brain barrier and cause changes in ICP in the postoperative period [9,10].

The average ICP range may vary with age. It is accepted as <10–15 mm Hg in adults, 3–7 mm Hg in children and 1.5–6 mm Hg in term newborns. However, the starting limits for treating intracranial hypertension also vary according to the etiology. Today, discussions continue about the standard upper limit of etiological causes. For example, several authors have recommended 15, 20 and 25 mm Hg values for the initiation of high ICP therapy in patients with head injuries [11].

An increase in intracranial pressure, whether the cause is neurologic or not, can cause bad results [11]. Therefore, early detection and management are essential. Intraventricular catheterization and intraparenchymal probes are accepted as the gold standard for ICP monitoring [12]. However, the invasive nature of these procedures and the high risk of complications prevent routine applications. Accordingly, non-invasive measurement methods for ICP have gained more importance with each passing day.

The first sign of increased ICP is optic disc enlargement [13]. Ophthalmoscopic evaluation may not be possible in all patients due to unconsciousness and injury in the eye area. In addition, an increase in ONSD is detected earlier than in ophthalmoscopic findings. While papilledema may take hours to days to develop, ONSD increase due to ICP increase occurs within a few minutes [14]. Compared to other diagnostic methods, the ultrasound measurement of ONSD is the fastest and easiest method to evaluate ICP increases. The direct visualization of the optic nerve is possible with ultrasound [15].

Optic nerve sonography is performed with a high-frequency linear transducer (>7.5 MHz) with the patient lying supine, head in a neutral position and both eyes closed. The probe is gently placed in an axial plane on the temporal side of the closed upper eyelid using a thick layer of sterile ultrasound gel. B mode is selected. A transverse sonographic section allows for the visualization of structures of the retro-bulbar area, including the globe and the longitudinal route of the optic nerve [16,17,18]. Traditionally, ONSD measurement is performed 3 mm posterior to the base of the papilla by placing a manual cursor over the outer contours of the optic nerve sheath [14,19].

Direct optic nerve imaging is possible with ultrasonography, a standard, inexpensive and non-invasive method that offers rapid bedside examinations in emergency, operating room and intensive care settings. The method was pioneered in the 1970s by Ossoinig, who used the A-scan technique to distinguish the optic nerve from the perineural sheath [18]. The ultrasonographic evaluation of optic nerve sheath diameter (ONSD) has been proposed as a non-invasive measure of intracranial pressure and was first described in 1987 [17].

Although a specific threshold value for optic nerve sheath diameter (ONSD) has yet to be determined in the literature, 5 mm has been accepted as the limit in most studies [16]. A study has shown that ICP is >20 mmHg when the ONSD exceeds 5 mm. If the ONSD breakpoint is >4.5 mm, the sensitivity is 100%, and the specificity is 63% in detecting the increase in ICP [20]. In a meta-analysis conducted in 2010, intracranial pressure (ICP) measurements performed with intraparenchymal or intraventricular applications, which are accepted as the gold standard, and USG and ONST measurements were compared, and no significant difference was found in terms of sensitivity, specificity, negative predictive or positive predictive value in both groups [21].

Optic nerve sheath diameter (ONSD) values are indirect predictive markers for ICP [22]. The most significant advantage of the procedure is that it can be performed quickly and easily with bedside ultrasonography. Studies have shown that the process is easily reproducible and has low inter-observer variability [23]. Considering that computed tomography (CT) or magnetic resonance imaging, which are other noninvasive methods, cannot be performed at the bedside, as well as the need for patient transfer and the time it takes due to this, ultrasonographic ONSD measurement may be an excellent alternative method for detecting ICP increases. There is currently no optimal ONSD cut-off value for an ICP increase. However, studies report the correlation between ONSD and ICP measurement values [24].

Using optic nerve ultrasonography or magnetic resonance imaging, different studies have demonstrated enlarged optic nerve sheaths in children and adults with elevated intracranial pressure due to various pathologies. In addition, they have shown that increased ONSD is a sensitive sign of intracranial hypertension [25,26].

In the literature, we could not find any study about ONSD measurement during extracorporeal circulation in the adult patient group. In this study, we aimed to evaluate the optic nerve sheath diameter measurements under the guidance of ultrasonography for ICP changes during the extracorporeal circulation process.

## 2. Materials and Methods

### 2.1. Ethics and Study Design

This study was carried out according to the Declaration of Helsinki or ethical rules after the ethics committee approval (decision no: 2022-KAEK-98) of the KastamonuTraining and Research Hospital. The study population was 21 patients over 18 who underwent coronary artery surgery and mitral or aortic valve replacement surgery requiring extracorporeal circulation. Written informed consent was obtained from all patients. Patients with known ophthalmic disease, a history of ophthalmic surgery and any known neurological disorder were excluded from the study. Demographic data such as age, gender, comorbidity, American Society of Anesthesiologists (ASA) classification and reason for operation (coronary artery disease and mitral or aortic valve disease) were recorded. ONSD and calculated patients’ non-invasive ICP (ICP ONSD) values were recorded.

### 2.2. ONSD Measurement

An anesthesiologist with experience in ocular sonography made all the measurements. Sonographic examinations were performed using a Sono Site X-Porte Fuji Film Sono Site (Inc., Bothell, WA, USA) equipped with a 13.5 MHz linear probe. Measurements were performed in the supine position with the patient’s eyes closed. The probe was gently placed on the upper eyelid with a standard ultrasound gel and adjusted at an appropriate angle to show the entrance of the optic nerve to the eyeball. The ONSD was measured bilaterally 3 mm behind the papilla. Two measurements, one for the transverse and one for the spine, were calculated for both optic nerves. The average of these two measurements was recorded for the final ounce measurement value. The ONSD was measured just before the start of the extracorporeal circulation (1st time) and at the 30th minute (2nd time), 60th minute (3rd time) and 90th minute (4th time) of the extracorporeal circulation. nICPON values calculated using the measured ONSD values were recorded according to the formula nICPON = 5 × ONSD − 14 (mm Hg) stated by Robba et al. [27]. The ONSD measurements were terminated at the 90th minute due to different extracorporeal circulation times and the limited patient population. The mean arterial pressure (MAP), saturation (SpO_2_), power of hydrogen value in arterial blood gas (Ph), partial oxygen pressure (pO_2_), partial carbon dioxide pressure (pCO_2_), and lactate values were recorded at the measurement times.

### 2.3. Statistical Analysis

The means and ranges were calculated for continuous variables. Numbers and percentages were calculated for categorical variables. The means and ranges were calculated for ONSD, nICPON, hemodynamic and respiratory variables at the four time points. A Wilcoxon signed-ranks test was used for ONSD measurements and the calculated nICPON values at the four time points. All statistical analyses were performed using SPSS Version 26.00 (SPSS Inc., Chicago, IL, USA). A *p*-value of <0.05 was considered statistically significant.

## 3. Results

The study cohort was made of 21 patients over 18 years of age who were operated on for coronary artery and mitral or aortic valve disease using extracorporeal circulation. The mean age of fifteen male and six female patients included in the study group was 63.24 (49.0–89.0) years. The most common comorbidity was hypertension. While the ASA physical status of 9 patients was III, the ASA physical status of the remaining 12 patients was IV. Fifteen patients underwent coronary artery surgery, and six underwent mitral or aortic valve replacement surgery. The demographic characteristics of the patients are shown in Table 1.

When the patients’ MAP, SpO_2_, Ph, pO_2_, pCO_2_ and lactate values at the four perioperative time points were measured, no statistically significant difference was found. Therefore, the MAP, SpO_2_, Ph, pO_2_, pCO_2_ and lactate values at the 0th, 30th, 60th, and 90th minutes of the perioperative period are given in Table 2.

The mean ONSD values measured before the extracorporeal circulation of the patients were found to be 4.13 mm (3.8–4.6) for the right eye and 4.36 mm (4.1–4.7) for the left eye. Calculated nICPONSD values for the right eye 11.0 mm Hg (1.0–21.0) and the left eye 10.89 mm Hg (1.0–21.0) were found. When the 30th-, 60th-, and 90th-minute ONSD measurements and calculated nICPONSD values were compared with the ONSD measurement values just before the extracorporeal circulation, the ONSD measurement and calculated nICPONSD values of the patients during the whole extracorporeal circulation for the right and left eyes were statistically significantly higher (Table 3).

## 4. Discussion

In our study, periodic ONSD measurements were made in 21 patients during extracorporeal circulation, and nICPONSD values were calculated. During the extracorporeal circulation process, both ONSD and nICPONSD values increased over time, and there was a statistical difference between the 0th, 30th, 60th and 90th minutes.

The gold standard of ICP measurement is the invasive technique, extra ventricular drainage (EVD). Non-invasive predicted ICP measurement techniques include ultrasound, magnetic resonance, and computed tomography measurements. The normal ICP value is 5–15, and values above 20 mmHg indicate increased ICP [28].

The optic nerve is part of the central nervous system and is surrounded by the dura, arachnoid and piamater. The optic nerve sheath is the continuation of the dura mater, and the subarachnoid space contains cerebrospinal fluid (CSF). Thus, any increase in ICP in the subarachnoid area is transferred to the fluid in the surrounding optic nerve [29]. As a result of this transferred pressure, the optic nerve sheath expands, and its diameter increases [30,31]. In a meta-analysis by Dubourg et al., which compared ONSD ultrasonography with ICP impression, including six studies involving 231 patients, it was emphasized that ONSD measurements showed excellent diagnostic accuracy in detecting intracranial hypertension and could help clinical decisions to be made [21]. Furthermore, Geeraerts et al. compared invasive ICP and ONSD measurements in 37 adult patients with severe traumatic brain injury, subarachnoid hemorrhage, intracranial hematoma, or post-stroke who required sedation and ICP monitoring. As a result, it has been reported that ultrasound-guided ONSD measurement in sedated neurocritical care patients followed in the intensive care unit is associated with invasive ICP and can be used as a screening test in patients with high ICP [16]. Newman et al. studied 23 children with shunted hydrocephalus and suggested increasing ICP increases ONSD values [23]. Tayal et al. compared the ONSD values of 59 adult patients with symptoms of increased intracranial pressure in the emergency department with computed tomography findings and found a high correlation. In conclusion, they emphasized that ONSD could be a sensitive screening test for increased intracranial pressure in adult head injuries [25]. Finally, Malayeri et al. compared the ONSD values of 156 children, 78 in the case group and 78 in the control group. They found that the ONSD values were significantly greater in pediatric patients with increased ICP compared with the control group [26].

Because patients cannot be taken for radiological imaging due to extracorporeal circulation, anticoagulant treatment for extracorporeal circulation prevents ICP measurement with invasive methods. Robba et al. reported in their study that non-invasive ICP (nICPONSD) estimated by ONSD can be calculated with the formula = 5 × ONSD − 14 (nICPONSD in mm Hg, ONSD in mm) [27]. In our study, nICPONSD values calculated from ONSD values showed a statistical increase compared to the baseline values, and no nICPONSD values exceeded the value expressing increased ICP.

The relationship between ONSD and ICP has been studied in many clinical situations. In the study by Rehman et al., ultrasound-guided ONSD measurements of 26 adult patients diagnosed with idiopathic intracranial hypertension and 26 regular healthy adult patients were evaluated, and it was emphasized that ultrasound-guided ONSD measurement is a reliable non-invasive method in the follow-up of patients with benign intracranial hypertension (BIH) [31]. Skoloudik et al. evaluated the computed tomography, ultrasound-accompanied ONSD values and color-coded duplex sonography findings of all patients in their study with 31 patients with acute intracerebral hemorrhage, 15 patients with acute ischemic stroke, and 16 healthy volunteers. As a result, they reported that increased intracranial pressure could be detected by ONSD measurement in the hyperacute period [32]. Strapazon et al. investigated the effects of hypobaric hypoxia on the ONSD and stated that both physiological and pathological responses to hypobaric hypoxia were independently associated with ONSD changes [33]. Lefferts et al. investigated the effects of the ONSD and ophthalmic arterial blood flow pulsatility in 20 participants in their study. They found no statistically significant change in ONSD values during recovery from acute high-intensity resistance exercise [34]. Dinsmore et al. examined the effects of acutely controlled changes in end-tidal carbon dioxide on the ONSD in 11 healthy volunteers. They showed that the ONSD changes rapidly with increases in end-tidal carbon dioxide. In conclusion, they emphasized that transorbital ultrasonography is a simple and non-invasive tool that can quickly and effectively measure acute and fluctuating changes in ICP in both fully awake and anesthetized patients [35]. Finally, Thakkar et al. divided 90 patients who underwent elective primary brain tumor resection into three groups of 30 people according to the surgical position and evaluated the ONSD values in these three different positions [36]. Robba et al., in a study of 30 patients undergoing spinal surgery, aimed to investigate the effects of prone position and positive end-expiratory pressure on the ONSD and reported that the ONSD might be helpful for ICP monitoring in patients requiring a prone position or at risk of developing hypertension [37].

We could not find any study investigating the relationship between ICP and ONSD in extracorporeal circulation in adults. In pediatric patients, Wakimoto et al. reported no change in optic nerve sheath diameters before and after CPB in children undergoing congenital heart surgery. In the subgroup of children with only one ventricle physiology, pre- and post-CPB ONDS values were statistically higher than in other congenital heart surgery operations. However, they also reported in their study that there were no clinical signs suggestive of ICP [38]. Currently, there is still no optimal ONSD cut-off value for an ICP increase. On the other hand, Shirodkar et al. found a threshold value of 5.7 mm in a study in which they looked at ONSD measurements in 100 adults followed up with meningoencephalitis in the intensive care unit [39]. In a study by Soldatos et al. involving 76 critical care patients (50 patients with brain damage and 26 patients in the control group), in the statistical analysis, they included the threshold value for increased intracranial pressure. Similar to the work of Shirodkar et al., they found it to be 5.7 mm [40]. In another study, in which they looked at the correlation of the ONSD with the direct measurement of intracranial pressure, Kimberly et al. evaluated the threshold value of the ONSD as 5 mm [20]. Lee et al., in their study involving 134 Korean patients, found the ounce threshold for increased ICP to be 5.5 mm [41]. Amini et al. measured the ONSD before procedures in 50 patients who were to undergo lumbar puncture and then measured and compared ICP with lumbar puncture and reported the ONSD threshold value as 5.5 mm [42].

ONSD values can also vary according to race. For example, the ONSD value was accepted as 4.41 mm in a study conducted with 15 healthy volunteers in Bangladesh, the ONSD value was accepted as 5.1 mm in a study conducted with 519 healthy volunteers in China, and the ONSD value was accepted as 4.11 mm in a study in which 585 healthy volunteers were included in Korea. The ONSD value was 5.95 mm in a study conducted with 20 healthy volunteers in Italy, the ONSD value was 4.6 mm in a study conducted with ten healthy volunteers in the United States, the ONSD value was 3.68 mm in a study conducted with 120 healthy volunteers in Canada, and the ONSD value was 4.86 mm in a study conducted with 160 healthy volunteers in Turkey [19,43,44,45,46,47]. However, studies reported the lowest limit of ONSD values as ≥5.2–5.3 mm in patients with high ICP [40,41,42]. Again, in the literature, nICPONSD values calculated from ONSD values were found to be 11.00 mm Hg (1.0–21.0) for the right eye and 10.89 mm Hg (1.0–21.0) for the left eye and did not exceed the lower value, indicating ICP increases [23].

In our study, the mean ONSD value was found to be 4.13 mm for the right eye and 4.23 mm for the left eye, similar to the literature. We found that 4 of 168 measurements had ONSDs ≥5.20 mm. While two of these measurements were 5.2 mm, the other two were 5.25 mm and below 5.3 mm, which is considered the upper value.

Oxygen and glucose consumption in the brain are closely related. Non-invasive methods can demonstrate cerebral blood flow (CBF). Hemodynamic instability, pH and temperature are the main factors affecting CBF during CPB [48]. The ability of cerebral perfusion to keep the total CBF constant despite changes in perfusion pressure in CBP is called ‘cerebral autoregulation.’ Under normal conditions, this autoregulation occurs through an active change in cerebral resistance. If intracranial pressure is normal, as long as the mean arterial blood pressure is between 60 and 150 mmHg, the CBF remains at 50 mL/min-100 g.

At the lower limit of autoregulation, cerebral vasodilation is at its maximum level, and CBF decreases with cerebral perfusion pressure when it falls further. When the upper limit of vasoconstriction is exceeded, the increase in vascular pressure and CBF may cause damage to the blood–brain barrier [6]. Although the brain is protected by autoregulation, it is vulnerable to injury in cardiac surgery. Autoregulation is affected by body temperature, blood flow pattern, viscosity, O_2_ and CO_2_ pressure, and pharmacological agents. In studies with experimental sections, it was determined that in animals, BB phosphokinase isoenzyme levels, which are essential indicators of non-pulsatile damage, increased significantly in pulsatile CPB compared to non-pulsatile CPB. In addition, cerebral capillary diameter narrowing has been observed more in non-pulsatile CPB, and it has been reported that this situation affects cerebral perfusion negatively. The consensus is that pulsatile CPB improves brain functions and cerebral blood flow rate [6]. Despite all this, the effect of CPB duration on the degree of deterioration of the blood–brain barrier remains unclear. Schuller et al. suggested that prolonged CPB duration induces a neutrophil-mediated reduction in β-catenin expression from cerebral microvascular endothelial cells. Consequently, a longer CPB duration results in moreblood–brain barrier damage [49].

Our study is the first to use the ONSD before and during extracorporeal circulation in an adult patient group. More than one factor may be responsible for the neurological damage and increased ICP saw after surgery for extracorporeal circulation and coronary heart disease. These factors can be patient-related as well as intraoperative factors. Factors associated with the patient include being over 60 years old, female gender, poor preoperative left ventricular function, critical preoperative condition, diabetes, hypertension, and previous stroke [50]. The mean age of our study cohort was 63.24, and the female gender ratio was 28.6%. Hypertension was observed in 33.3% of the patients, and diabetes was observed in 28.6%. The patients in our study underwent a single surgical intervention. Both coronary artery and mitral–aortic valve patients had higher ONSD values during extracorporeal circulation than pre-extracorporeal circulation ONSD values. It was observed that there was an increase in ONSD values with the extracorporeal circulation time. This may have been due to intraoperative risk factors. Cross-clamping on aortic atheroma, microembolization during CPB, hypoperfusion, and temperature variables are also considered among intraoperative risk factors [50,51]. An increase in ICP values may be observed depending on these reasons. However, all of our measurements were below the ONSD measurement values, indicating ICP increases.

The limitations of this study are that it was single-center, included a small study size, and the ONSD measurement was limited to 90 min. Another limitation is that it did not evaluate the relationship of ONSD values during extracorporeal circulation with postoperative clinical outcomes.

## 5. Conclusions

In conclusion, ultrasound-guided ONSD measurement is an easy, inexpensive, and low-complication method that can be performed at the bedside during an operation to monitor ICP changes in patients in the extracorporeal circulation process. In our study, all ONSD measurement and nICPONSD values in the extracorporeal circulation process were higher than the initial ONSD measurement values. More general conclusions can be reached with reflections on this subject with large patient populations and longer patient follow-ups.

## Figures and Tables

**Table 1:** Demographic characteristics of the patients.

		All Patients (*n* = 21)
Age		63.24 (49.0–89.0)
Gender	Male	15 (71.4%)
	Female	6 (28.6%)
Additional Disease	None	2 (9.5%)
	Diabetes Mellitus	6 (28.6%)
	Respiratory Disease	5 (23.8%)
	Renal Failure	1 (4.8%)
	Hypertension	7 (33.3%)
ASA	3	12(57.1%)
	4	9 (42.9%)

ASA: American Society of Anesthesiologists.

**Table 2:** Patients’ perioperative MAP, SpO_2_, Ph, pO_2_, pCO_2_ and lactate values at four time points.

All Patients (*n* = 21)	0 min	30 min	60 min	90 min
Mean Arterial Pressure	76.43 (65.0–85.0)	57.0 (48.0–70.0)	57.14 (49.0–63.0)	61.29 (47.0–70.0)
SpO_2_	99.57 (99.0–100)	99.57 (99.0–100)	98.9 (97.0–100)	99.43 (99.0–100)
Ph	7.38 (7.3–7.4)	7.32 (7.25–7.38)	7.33 (7.29–7.42)	7.32 (7.27–7.32)
pO_2_	193.86 (156.0–239.0)	279.48 (195.0–355.0)	247.24 (189.0–298.0)	270.62 (211.0–313.0)
pCO_2_	38.07 (29.0–42.6)	38.92 (33.0–44.8)	33.81 (24.0–42.0)	35.12 (27.0–42.6)
Lactic Acid Value	1.11 (0.6–1.4)	1.94 (1.2–2.7)	2.64 (1.4–3.7)	3.57 (1.9–5.5)

**Table 3:** ONSD and nICPONSD measurements and extracorporeal circulation time.

	Time	Right Eye	*p*	Left Eye	*p*
ONSD	0 min 30 min	4.13 (3.8–4.6) 4.36 (4.1–4.7)	<0.001	4.23 (3.8–4.5) 4.39 (4.15–4.7)	0.003
nICPONSD	0 min 30 min	6.67 (5.00–9.00) 7.83 (6.50–9.50)	<0.001	7.19 (5.00–8.50) 7.97 (6.75–9.50)	0.001
ONSD	0 min 60 min	4.13 (3.8–4.6) 4.63 (4.20–5.0)	<0.001	4.23 (3.8–4.5) 4.58 (4.3–4.9)	<0.001
nICPONSD	0 min 60 min	6.67 (5.00–8.50) 9.17 (7.00–11.00)	<0.001	7.19 (5.00–8.50) 8.91 (7.50–10.50)	<0.001
ONSD	0 min 90 min	4.13 (3.8–4.6) 4.99 (4.7–5.25)	<0.001	4.23 (3.8–4.5) 4.88 (4.5–5.25)	<0.001
nICPONSD	0 min 90 min	6.67 (5.00–8.50) 10.96 (8.5–12.25)	<0.001	7.19 (5.00–8.50) 10.44 (8,501,225)	<0.001

ONSD: optic nerve sheath diameter; nICPONSD: non-invasive ICP.

## Data Availability

Not applicable.

## References

[1] Bilal M.S., Sarıoğlu T. (1992). İskemikmiyokardinjurisiveintraoperatifmiyokardkorunmasınagenelbirbakış. Türk. Göğüs. Kalp. Damar. Cerrahisi. Dergisi..

[2] Barlett H., Delius R.E., Kay P.H. (1992). Phsiology and Pathophysiology of Extracorporeal Circulation. Technigues in Extracorporeal Circulation.

[3] Bigi L., Guelli N., Menghini A., Panjani I., Kay P.H. (1992). Design and Principles of the Extracorporeal Circuit. Technigues in Extracorporeal Circulation.

[4] Machin D., Allsager C. (2006). Principles of cardiopulmonary bypass. Contin. Educ. Anaesth. Crit. Care Pain.

[5] Gaynor J.W., Stopp C., Wypij D., Andropoulos D.B., Atallah J., Atz A.M., Beca J., Donofrio M.T., Duncan K., Ghanayem N.S. (2015). Neurodevelopmental outcomes after cardiac surgery in infancy. Pediatrics.

[6] Murkin J.M., Farrar J.K., Tweed W.A., McKenzie F.N., Guiraudon G. (1987). Cerebral autoregulation and flow/metabolism coupling during cardiopulmonary bypass: The influence of PaCO2. Anesth. Analg..

[7] Kussman B.D., Wypij D., Laussen P.C., Soul J.S., Bellinger D.C., DiNardo J.A., Robertson R., Pigula F.A., Jonas R.A., Newburger J.W. (2010). Relationship of intraoperative cerebral oxygen saturation to neurodevelopmental outcome and brain magnetic resonance imaging at 1 year of age in infants undergoing biventricular repair. Circulation.

[8] Hansen J.H., Rotermann I., Logoteta J., Jung O., Dütschke P., Scheewe J., Kramer H.H. (2016). Neurodevelopmental outcome in hypoplastic left heart syndrome: Impact of perioperative cerebral tissue oxygenation of the Norwood procedure. J. Thorac. Cardiovasc. Surg..

[9] Abrahamov D., Levran O., Naparstek S., Refaeli Y., Kaptson S., Abu Salah M., Ishai Y., Sahar G. (2017). Blood-Brain Barrier Disruption After Cardiopulmonary Bypass: Diagnosis and Correlation to Cognition. Ann. Thorac. Surg..

[10] Cavaglia M., Seshadri S.G., Marchand J.E., Ochocki C.L., Mee R.B., Bokesch P.M. (2004). Increased transcription factor expression and permeability of the blood brain barrier associated with cardiopulmonary bypass in lambs. Ann. Thorac. Surg..

[11] Dunn L.T. (2002). Raised intracranial pressure. J. Neurol. Neurosurg. Psychiatry.

[12] Raboel P.H., Bartek J., Andresen M., Bellander B.M., Romner B. (2012). Intracranial Pressure Monitoring: Invasive versus Non-Invasive Methods-A Review. Crit. Care Res. Pract..

[13] Hayreh M.S., Hayreh S.S. (1977). Optic disc edema in raised intracranial pressure. I. Evolution and resolution. Arch. Ophthalmol..

[14] Hansen H.C., Helmke K. (1997). Validation of the optic nerve sheath response to changing cerebrospinal fluid pressure: Ultrasound findings during intrathecal infusion tests. J. Neurosurg..

[15] Blaivas M., Theodoro D., Sierzenski P.R. (2003). Elevated intracranial pressure detected by bedside emergency ultrasonography of the optic nerve sheath. Acad. Emerg. Med..

[16] Geeraerts T., Merceron S., Benhamou D., Vigué B., Duranteau J. (2008). Non-invasive assessment of intracranial pressure using ocular sonography in neurocritical care patients. Intensive Care Med..

[17] Gangemi M., Cennamo G., Maiuri F., D’Andrea F. (1987). Echographic measurement of the optic nerve in patients with intracranial hypertension. Neurochirurgia.

[18] Ossoinig K.C. (1979). Standardized echography: Basic principles, clinical applications, and results. Int. Ophthalmol. Clin..

[19] Maude R.R., Hossain M.A., Hassan M.U., Osbourne S., Sayeed K.L., Karim M.R., Samad R., Borooah S., Dhillon B., Day N.P. (2013). Transorbital sonographic evaluation of normal optic nerve sheath diameter in healthy volunteers in Bangladesh. PLoS ONE.

[20] Kimberly H.H., Shah S., Marill K., Noble V. (2008). Correlation of optic nerve sheath diameter with direct measurement of intracranial pressure. Acad. Emerg. Med..

[21] Dubourg J., Javouhey E., Geeraerts T., Messerer M., Kassai B. (2011). Ultrasonography of optic nerve sheath diameter for detection of raised intracranial pressure: A systematic review and meta-analysis. Intensive Care Med..

[22] Romagnuolo L., Tayal V., Tomaszewski C., Saunders T., Norton H.J. (2005). Optic nerve sheath diameter does not change with patient position. Am. J. Emerg. Med..

[23] Newman W.D., Hollman A.S., Dutton G.N., Carachi R. (2002). Measurement of optic nerve sheath diameter by ultrasound: A means of detecting acute raised intracranial pressure in hydrocephalus. Br. J. Ophthalmol..

[24] Bäuerle J., Lochner P., Kaps M., Nedelmann M. (2012). Intra- and interobsever reliability of sonographic assessment of the optic nerve sheath diameter in healthy adults. J. Neuroimaging.

[25] Tayal V.S., Neulander M., Norton H.J., Foster T., Saunders T., Blaivas M. (2007). Emergency department sonographic measurement of optic nerve sheath diameter to detect findings of increased intracranial pressure in adult head injury patients. Ann. Emerg. Med..

[26] Malayeri A.A., Bavarian S., Mehdizadeh M. (2005). Sonographic evaluation of optic nerve diameter in children with raised intracranial pressure. J. Ultrasound Med..

[27] Robba C., Donnelly J., Cardim D., Tajsic T., Cabeleira M., Citerio G., Pelosi P., Smielewski P., Hutchinson P., Menon D.K. (2019). Optic nerve sheath diameter ultrasonography at admission as a predictor of intracranial hypertension in traumatic brain injured patients: A prospective observational study. J. Neurosurg..

[28] Nag D.S., Sahu S., Swain A., Kant S. (2019). Intracranial pressure monitoring: Gold standard and recent innovations. World J. Clin. Cases.

[29] Killer H.E., Laeng H.R., Flammer J., Groscurth P. (2003). Architecture of arachnoid trabeculae, pillars, and septa in the subarachnoid space of the human optic nerve: Anatomy and clinical considerations. Br. J. Ophthalmol..

[30] Ballantyne J., Hollman A.S., Hamilton R., Bradnam M.S., Carachi R., Young D.G., Dutton G.N. (1999). Transorbital optic nerve sheath ultrasonography in normal children. Clin. Radiol..

[31] Rehman H., Khan M.S., Nafees M., Rehman A.U., Habib A. (2016). Optic Nerve Sheath Diameter on Sonography in Idiopathic Intracranial Hypertension Versus Normal. J. Coll. Physicians Surg.-Pak. JCPSP.

[32] Skoloudík D., Herzig R., Fadrná T., Bar M., Hradílek P., Roubec M., Jelínková M., Sanák D., Král M., Chmelová J. (2011). Distal enlargement of the optic nerve sheath in the hyperacute stage of intracerebral haemorrhage. Br. J. Ophthalmol..

[33] Strapazzon G., Brugger H., Dal Cappello T., Procter E., Hofer G., Lochner P. (2014). Factors associated with optic nerve sheath diameter during exposure to hypobaric hypoxia. Neurology.

[34] Lefferts W.K., Hughes W.E., Heffernan K.S. (2015). Effect of acute high-intensity resistance exercise on optic nerve sheath diameter and ophthalmic artery blood flow pulsatility. J. Hum. Hypertens..

[35] Dinsmore M., Han J.S., Fisher J.A., Chan V.W.S., Venkatraghavan L. (2017). Effects of acute controlled changes in end-tidal carbon dioxide on the diameter of the optic nerve sheath: A transorbital ultrasonographic study in healthy volunteers. Anaesthesia.

[36] Thakkar K.D., Sethuraman M., Praveen C.S.R., Vimala S., Hrishi P.A.P., Prathapadas U. (2022). Effect of Different Surgical Positions on the Changes in Cerebral Venous Drainage in Patients Undergoing Neurosurgery: A Prospective Observational Study. J. Neurosurg. Anesthesiol..

[37] Robba C., Bragazzi N.L., Bertuccio A., Cardim D., Donnelly J., Sekhon M., Lavinio A., Duane D., Burnstein R., Matta B. (2017). Effects of Prone Position and Positive End-Expiratory Pressure on Noninvasive Estimators of ICP: A Pilot Study. J. Neurosurg. Anesthesiol..

[38] Wakimoto M., Patrick J.H., Yamaguchi Y., Roth C., Corridore M., Tobias J.D. (2022). Optic nerve ultrasound and cardiopulmonary bypass: A pilot study. Saudi J. Anaesth..

[39] Shirodkar C.G., Munta K., Rao S.M., Mahesh M.U. (2015). Correlation of measurement of optic nerve sheath diameter using ultrasound with magnetic resonance imaging. Indian J. Crit. Care Med..

[40] Soldatos T., Karakitsos D., Chatzimichail K., Papathanasiou M., Gouliamos A., Karabinis A. (2008). Optic nerve sonography in the diagnostic evaluation of adult brain injury. Crit. Care.

[41] Lee S.U., Jeon J.P., Lee H., Han J.H., Seo M., Byoun H.S., Cho W.S., Ryu H.G., Kang H.S., Kim J.E. (2016). Optic nerve sheath diameter threshold by ocular ultrasonography for detection of increased intracranial pressure in Korean adult patients with brain lesions. Medicine.

[42] Amini A., Kariman H., Arhami Dolatabadi A., Hatamabadi H.R., Derakhshanfar H., Mansouri B., Safari S., Eqtesadi R. (2013). Use of the sonographic diameter of optic nerve sheath to estimate intracranial pressure. Am. J. Emerg. Med..

[43] Chen H., Ding G.S., Zhao Y.C., Yu R.G., Zhou J.X. (2015). Ultrasound measurement of optic nerve diameter and optic nerve sheath diameter in healthy Chinese adults. BMC Neurol..

[44] Kim D.H., Jun J.S., Kim R. (2017). Ultrasonographic measurement of the optic nerve sheath diameter and its association with eyeball transverse diameter in 585 healthy volunteers. Sci. Rep..

[45] Lochner P., Coppo L., Cantello R., Nardone R., Naldi A., Leone M.A., Brigo F. (2014). Intra- and interobserver reliability of transorbital sonographic assessment of the optic nerve sheath diameter and optic nerve diameter in healthy adults. J. Ultrasound.

[46] Zeiler F.A., Ziesmann M.T., Goeres P., Unger B., Park J., Karakitsos D., Blaivas M., Vergis A., Gillman L.M. (2016). A unique method for estimating the reliability learning curve of optic nerve sheath diameter ultrasound measurement. Crit. Ultrasound J..

[47] Gökçen E., Çaltekin İ., Albayrak L., Savrun A., Atik D., Vural S., Kılıç N., Kuşdoğan M., Kaya H.B. (2021). Assesment of OpticNerve Sheath Diameter in Healthy Adults in Turkey. Sakarya Tıp. Dergisi..

[48] Sarkar M., Prabhu V. (2017). Basics of cardiopulmonary bypass. Indian J. Anaesth..

[49] Schuller A.M., Windolf J., Blaheta R., Cinatl J., Kreuter J., Wimmer-Greinecker G., Moritz A., Scholz M. (2005). Degradation of microvascular brain endothelial cell beta-catenin after co-culture with activated neutrophils from patients undergoing cardiac surgery with prolonged cardiopulmonary bypass. Biochem. Biophys. Res. Commun..

[50] Anyanwu A.C., Filsoufi F., Salzberg S.P., Bronster D.J., Adams D.H. (2007). Epidemiology of stroke after cardiac surgery in the current era. J. Thorac. Cardiovasc. Surg..

[51] Costa M.A., Gauer M.F., Gomes R.Z., Schafranski M.D. (2015). Risk factors for perioperative ischemic stroke in cardiac surgery. Rev. Bras. Cir. Cardiovasc..

